# Biosynthesis of *S*-adenosyl-methionine enhances aging-related defects in *Drosophila* oogenesis

**DOI:** 10.1038/s41598-022-09424-1

**Published:** 2022-04-04

**Authors:** Yoshiki Hayashi, Soshiro Kashio, Kazutoshi Murotomi, Shinjiro Hino, Woojin Kang, Kenji Miyado, Mitsuyoshi Nakao, Masayuki Miura, Satoru Kobayashi, Masakazu Namihira

**Affiliations:** 1 Life Science Center for Survival Dynamics, Tsukuba Advanced Research Alliance (TARA), University of Tsukuba, 1-1-1, Tennodai, Tsukuba, Ibaraki 305-8577 Japan; 2 Department of Genetics, Graduate School of Pharmaceutical Science, The University of Tokyo, 7-3-1, Hongo, Bunkyo-Ku, Tokyo 113-0033 Japan; 3 Biomedical Research Institute, National Institute of Advanced Industrial Science and Technology, 1-1-1, Higashi, Tsukuba, Ibaraki 305-8566 Japan; 4 Institute of Molecular Embryology and Genetics, Kumamoto University, 2-2-1, Honjo, Chuo-ku, Kumamoto 860-0811 Japan; 5 Department of Reproductive Biology, National Research Institute for Child Health and Development, 2-10-1, Okura, Setagaya-Ku, Tokyo 157-8535 Japan

**Keywords:** Ageing, Metabolomics

## Abstract

Tissue aging is a major cause of aging-related disabilities and a shortened life span. Understanding how tissue aging progresses and identifying the factors underlying tissue aging are crucial; however, the mechanism of tissue aging is not fully understood. Here we show that the biosynthesis of *S*-adenosyl-methionine (SAM), the major cellular donor of methyl group for methylation modifications, potently accelerates the aging-related defects during *Drosophila* oogenesis. An aging-related increase in the SAM-synthetase (*Sam-S*) levels in the germline leads to an increase in ovarian SAM levels. *Sam-S*-dependent biosynthesis of SAM controls aging-related defects in oogenesis through two mechanisms, decreasing the ability to maintain germline stem cells and accelerating the improper formation of egg chambers. Aging-related increases in SAM commonly occur in mouse reproductive tissue and the brain. Therefore, our results raise the possibility suggesting that SAM is the factor related to tissue aging beyond the species and tissues.

## Introduction

**Figure 1:**
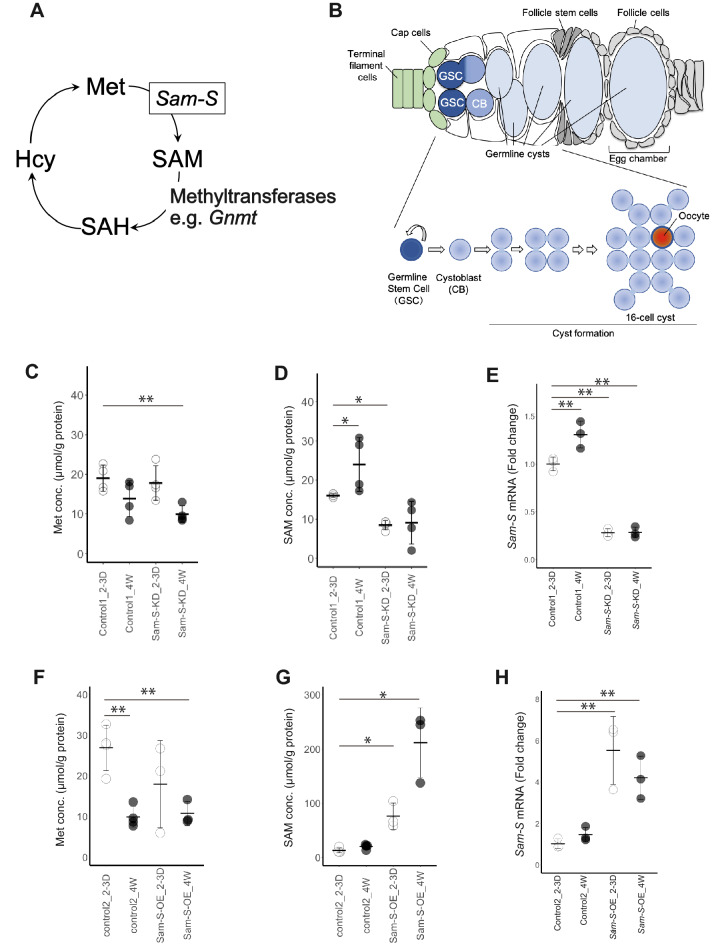
Aging-related change in SAM biosynthesis in *Drosophila* ovaries. (**A**) Schematic of Met metabolism. SAM is synthesised from Met by *Sam-S* activity. SAM is catabolised by methyltransferases, such as *Gnmt*, to provide a methyl group for methylation modification; SAM is converted to SAH and eventually to homocysteine (Hcy). The level of Hcy was below the detection limit. (**B**) Schematic of germline development within the germarium. By associating with somatic niche (terminal filament cells, cap cells, green in upper panel), 2–3 germline stem cells (GSCs, deep blue) were maintained. Asymmetric division of GSCs results in differentiating daughter cells called cystoblasts (CBs, light blue). CBs undergo mitosis four times with incomplete cytokinesis, giving rise to germline cysts with 16 interconnected germline cells (light blue in lower panel). Within each germline cyst, a germline cell with four cytoplasmic bridges becomes an oocyte (red in lower panel) and the others exist as nurse cells to support maturation of the oocyte. (**C**–**E**) Changes in the levels of Met, SAM, and *Sam-S* mRNA in control1 (See Materials and Methods) and *Sam-S* knocked-down (*Sam-S*-KD) ovaries in the respective age groups. *Sam-S* expression and SAM levels increased significantly with the ovarian age; the age-dependent increase of *Sam-S* and SAM were inhibited by germline knockdown of *Sam-S*. For quantification of metabolites, four samples from each genotype and age were examined. For quantification of *Sam-S* mRNA expression, three samples from each genotype and age were examined. (**F**–**H**) Changes in the levels of Met, SAM, and *Sam-S* mRNA in control2 (See Materials and Methods) and *Sam-S* overexpressed (*Sam-S*-OE) ovaries in the respective age groups. *Sam-S* expression and SAM levels significantly increased with the germline specific overexpression of *Sam-S*. For quantification of metabolites and *Sam-S* mRNA expression, three samples from each genotype and age were examined. For multiple comparison analyses in (**C**–**H**), statistical significance was calculated using Dunnett’s test by using the young control ovaries as the controls; *indicates *p* < 0.1, and **indicates *p* < 0.05. The Gal4-driver used in (**C**–**H**) was *nos*-Gal4. 2-3D; 2–3 days after eclosion, 4 W; 4 w after eclosion.

SAM is an essential metabolite and is the main cellular source of methyl groups for the methylation of proteins and nucleic acids; it has a crucial role in the regulation of the epigenomic status and gene expression. SAM is synthesised from Met and adenosine triphosphate (ATP) by the evolutionally conserved enzyme, Sam-S, also known as methionine adenosyltransferase (MAT) (Fig. 1A). SAM is catabolised by a variety of methyltransferases to *S*-adenosyl-homocysteine (SAH), which is eventually hydrolysed to adenosine and homocysteine (Fig. 1A). In worms, knockdown of *sams1*, the worm orthologue of *Sam-S,* extends the life span^1^. In fruit flies, the systemic SAM levels increase with age; enhancing SAM catabolism using glycine *N*-methyltransferase (Gnmt) extends the life span^2^. A growing body of evidence shows a strong relationship between methylation and aging of the cell or tissue^3^. Despite the importance of SAM metabolism in the regulation of life span, the influence of SAM biosynthesis in tissue aging is poorly demonstrated, because of the absence of a suitable model in which SAM-dependent aging-related events can be quantitatively analysed.

The *Drosophila* reproductive system represents an ideal model for studying tissue aging because of the short life span of *Drosophila* and the availability of well-established, powerful genetic tools and a large body of literature on tissue development. *Drosophila* females have a pair of ovaries that contain approximately 16 ovarioles. At the anterior tips of each ovariole, 2–3 GSCs are maintained by niche, named cap cells and terminal filament cells^4,5^ (Fig. 1B). GSCs divide asymmetrically to self-renew and provide differentiating daughter cells named cystoblasts (CBs). CBs undergo four mitotic divisions with incomplete cytokinesis and form germline cysts with 16 inter-connected germline cells. Each germline cyst is encapsulated by a monolayer of somatic follicular cells to form the egg chambers. Within the egg chambers, one of the two germline cells that have four cytoplasmic bridges is selected as an oocyte, and the remaining 15 germline cells exist as nurse cells to support the maturation of the oocyte (Fig. 1B). The study of *Drosophila* oogenesis can serve as a simple strategy to quantitatively investigate how aging affects tissue function because each of the above developmental events is easily quantifiable using a conventional fluorescent microscope. Indeed, it has been shown that the ability to maintain ovarian GSCs declines with age, partially due to oxidative stress in the GSCs and their stromal cells^6^. In this study, we investigated whether aging-related changes in SAM biosynthesis influence aging-related defects in oogenesis using *Drosophila* as a model.

## Results and discussion

We first investigated if the ageing-related change in SAM biosynthesis occurs during the aging of ovaries. We quantified the metabolites involved in SAM biosynthesis within the ovaries of young and aged females using ultra-performance liquid chromatography tandem mass spectrometry (UPLC-MS/MS). The SAM content increased approximately 1.5-fold in aged ovaries [mean ± standard deviation; Control1(#36,303 from BDSC, control for *Sam-S* RNAi): 16.06 ± 0.48 µM/g protein in young ovaries; 24.00 ± 6.90 µM/g protein in aged ovaries; Control2(#24,486 from BDSC, control for pUASp-*Sam-S*): 13.15 ± 4.64 µM/g protein in young ovaries; 20.13 ± 4.52 µM/g protein in aged ovaries. For detailed genotype information about control strains, please also refer to the material & method section) (Fig. 1D,G). Conversely, the content of the SAM precursor, Met, decreased by one-third in aged ovaries (Control1: mean ± standard deviation; 19.01 ± 3.31 µM/g protein in young ovaries; 13.84 ± 4.51 µM/g protein in aged ovaries; Control2: 26.91 ± 5.59 µM/g protein in young ovaries; 9.91 ± 2.56 µM/g protein in aged ovaries) (Fig. 1C,F). Therefore, we hypothesised that SAM biosynthesis was enhanced during the aging of ovaries. Using reverse transcription quantitative polymerase chain reaction (RT-qPCR), we confirmed that the expression of *Sam-S* was increased approximately 1.4-fold in aged ovaries (fold change in *Sam-S* mRNA expression between young and aged ovaries: mean ± standard deviation; Control1: 1.31 ± 0.14, Control2: 1.46 ± 0.35) (Fig. 1E,H). Therefore, SAM biosynthesis was enhanced in aged ovaries, suggesting that the aging-related increase in SAM biosynthesis could be one of the essential causal factors in the aging-related defects during oogenesis.

**Figure 2:**
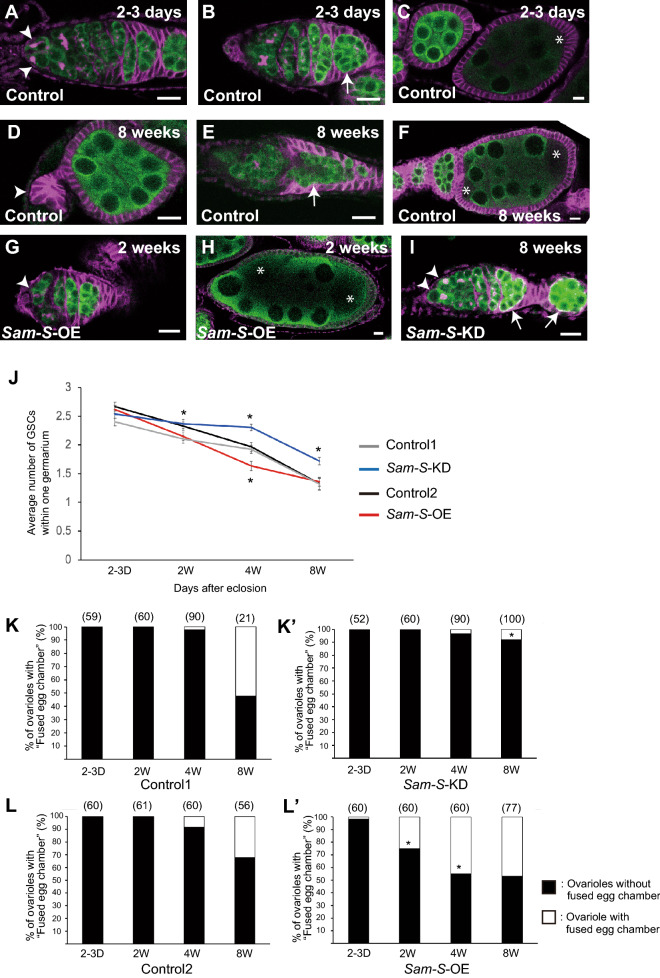
Relationship between SAM biosynthesis and age-related oogenesis defects. (**A**–**C**) Ovaries from young female flies (2–3 d after eclosion). In young ovaries, GSCs were maintained at the tips of each ovariole (Arrowheads in **A**), and germline cysts (Arrow in **B**) and egg chambers (**C**) were formed properly. Asterisk in (** C**) indicates oocyte within normal egg chamber. Oocytes are germline cells without large polyploid nuclei and that emit a weak Vasa signal. (**D**–**F**) Ovaries from aged female flies. In aged ovaries, the number of GSCs decreased; some ovarioles lacked GSCs (Arrowhead in **D**). Aged ovaries showed additional age-related defects, such as the improper formation of egg chambers (**E**,**F**). In aged ovaries, failed germline cyst encapsulation was observed during early oogenesis (**E**). These defective germline cysts resulted in egg chambers containing more than 16 germ cells, typically 32 germ cells with 2 oocytes (**F**). Asterisks indicate oocytes. (**G**,**H**) Ovaries of *Sam-S* overexpressed 2-week-old females. *Sam-S* overexpressed ovaries showed a decrease in GSCs (**G**, Arrowhead indicates GSC) and improper formation of the egg chamber (**H**, Asterisks indicate oocyte) even in middle age. (**I**) Ovaries from *Sam-S* knocked-down 8 week-old females. *Sam-S* knocked-down ovaries contained a relatively normal number of GSCs (indicated by arrowheads) and properly formed egg chambers (indicated by arrows). In (**A**–**I**), the green signal indicates Vasa protein, the germline marker, and the magenta signal indicates the Hu-li-tai-shao (Hts) protein that localises to the actin-rich cytoskeleton. Within germline cells in the germarium, Hts protein specifically localizes to a germline-specific organelle called fusome. Scale bars indicate 10 µm. (**J**) Age-related change in GSC number within an ovariole of the control (Control1; Grey, Control2; Black) and *Sam-S* genetically manipulated females (*Sam-S*-KD; Blue, *Sam-S*-OE; Red). Asterisks indicate statistical significance (*p* < 0.05) calculated using Student’s *t*-test. The following numbers of ovariole examined. Control1; 2–3 days: 60, 2 weeks: 60, 4 weeks: 90, 8 weeks: 38, *Sam-S*-KD; 2–3 days: 52, 2 weeks: 60, 4 weeks: 89, 8 weeks: 100, Control2; 2–3 days: 60, 2 weeks: 61, 4 weeks: 60, 8 weeks: 56, *Sam-S*-OE; 2–3 days: 60, 2 weeks: 60, 4 weeks: 60, 8 weeks: 90. (**K**–**L**’) Percentage of ovarioles showing “fused egg chamber phenotype” in the control (**K**; Control1, **L**; Control2) and *Sam-S* genetically manipulated ovaries (**K**’; *Sam-S*-KD, **L**’; *Sam-S*-OE). White bars indicate the percentage of ovarioles with the fused egg chamber phenotype. Asterisks in (**K**–**L**’) indicate statistical significance (*p* < 0.05) calculated using Fisher’s exact probability test. The numbers of ovariole examined is shown in parentheses.

Next, we investigated whether the increase in SAM biosynthesis causes aging-related defects in oogenesis. We confirmed the decline in the GSC number with age, as previously reported^6^. We counted the number of GSCs in the ovarioles at the respective ages and confirmed that it decreased with age in the control strains (Fig. 2A,D,J).

Furthermore, we found aging-related defects in the egg chamber formation. In the ovaries of young female flies, the germline cysts, consisting 16 interconnected germline cells, were encapsulated by follicular somatic cells and they appeared as egg chambers in the posterior end of the germaria (Fig. 2B, Arrow). These egg chambers were pinched off from the germaria and undergo further gametogenesis (Fig. 2C). However, we observed improper formation of egg chambers in the germaria and at later stages of oogenesis in old females (Fig. 2E,F, respectively). In the old ovarioles, germline cysts failed to be encapsulated by follicular cells and appeared as an egg chamber containing more than 16 germline cells; typically, 32 germline cells with two oocytes exist within one egg chamber (Fig. 2F). These observations suggest that egg chamber formation occurred improperly, resulting in an egg chamber containing two germline cysts in aged ovaries. We counted the number of ovarioles with egg chambers containing more than 16 germline cysts (here after, referred to as “fused egg chamber phenotype”); this phenotype was presented in females from the age of 4 w, the number of such individuals increased substantially at 8 w after eclosion (Fig. 2K,L). To note, we observed this age-related defect in fly strains of two independent genetic background (#36,303 and #24,486 from BDSC), suggesting that this age-related defect could occur in a variety of genetic backgrounds. Thus, these results suggest that aging affects egg chamber formation and that the egg chamber defect rate is an important indicator of aging-related defects during oogenesis.

To test if changes in SAM biosynthesis regulate the aging-related defects in oogenesis, we investigated whether the genetic manipulations of *Sam-S* cause aging-related defects in oogenesis. We used the Gal4–upstream activation sequence (UAS) system to overexpress or knockdown *Sam-S*. Aging-related defects in oogenesis were observed only in the germline but not in somatic ovarian cells; therefore, we used *nanos* (*nos*)-Gal4, a germline-specific Gal4 driver, to overexpress or knockdown *Sam-S* (UAS-*Sam-S* or UAS-*Sam-S* RNA interference (UAS-*Sam-S* RNAi), respectively). To evaluate whether these genetic manipulations effectively changed *Sam-S* mRNA expression and SAM levels in the ovaries, we compared these parameters between the control and genetically manipulated ovaries. First, we compared the *Sam-S* mRNA expression and SAM levels among the young females. Both the mRNA and metabolite levels were altered depending on the genetic manipulation of *Sam-S*, indicating that the genetic manipulations were effective (Fig. 1D,E,G,H). Next, we compared the same parameters between the young and aged *Sam-S*-knockdown ovaries and found that *Sam-S* knockdown repressed the age-dependent increase in *Sam-S* mRNA and SAM levels in the aged ovaries (Fig. 1D,E). These results indicate that the age-dependent increase in SAM biosynthesis in the ovaries mainly occurs in the germline and not in the somatic ovarian cells. *Sam-S* overexpression enhanced the age-dependent increase in SAM (Fig. 1G). *Sam-S* mRNA expression in the overexpressed ovaries did not increase during aging (Fig. 1H). This lack of change could be attributed to factors, such as the change in the usage of SAM. The amount of SAH—a catabolised metabolite of SAM—tended to decrease with age in all of the genetic combinations tested (Fig. S1A, B). Therefore, the genetically manipulated ovaries in this study are ideal models to observe the influence of SAM biosynthesis on the aging-related defects in oogenesis.

Our genetic manipulation of *Sam-S* in the germline successfully regulated SAM biosynthesis in the ovaries; therefore, we investigated whether this alternation in SAM biosynthesis affected age-related defects in oogenesis. First, we examined whether the changes in SAM biosynthesis affected the age-dependent decrease in the GSC maintenance ability. The age-dependent decrease in the GSC maintenance ability was enhanced in *Sam-S*-overexpressing ovaries; in contrast, it was suppressed in *Sam-S*-knockdown ovaries (Fig. 2G,I,J). Therefore, the age-related augmentation of SAM biosynthesis was one of the causal factors for the decreased GSC maintenance ability in ovaries from aged animals. Next, we investigated if this could be associated with the egg chamber formation defects in the aged ovaries. Age-related increases in the fused egg chamber phenotype were enhanced in *Sam-S*-overexpressing ovaries; however, similar to the observations related to GSC maintenance, this was conversely moderated in *Sam-S* knockdown ovaries (Fig. 2H,I,K–L’). Additionally, we investigated if the increase of SAM biosynthesis affects the other reported age-related germline defects, the germline apoptosis within the germarium^7^. We found that over-expression of *Sam-S* significantly increase the germline apoptosis within the germarium (Fig. S2A,B). Therefore, age-related enhancements in SAM biosynthesis were an important causal factor for the aging of oogenesis.

**Figure 3:**
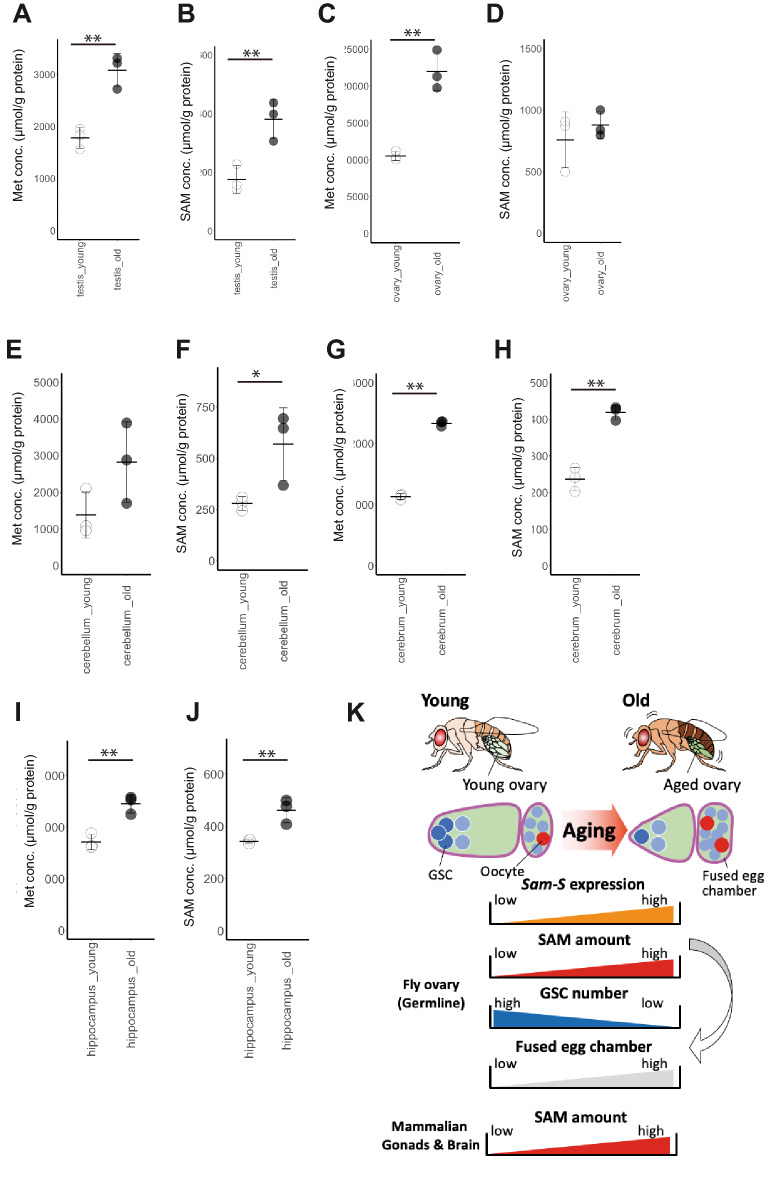
Age-related changes in Met metabolism in mouse tissues. (**A**–**J**) Comparisons of age-related changes in the Met and SAM levels in mouse reproductive tissues (**A**,**B**; Testis, **C**,**D**; Ovaries), and parts of brains (**E**,**F**; Cerebellum, **G**,**H**; Cerebrum, and **I**,**J**; Hippocampus). Both Met and SAM levels increased during aging in all the tissues investigated. For quantification of metabolites from mouse tissues, three samples from each tissue in respective age were examined. In all the panels, statistical significance was calculated using Weltch’s *t*-test; *indicates *p* < 0.1, and **indicates *p* < 0.05. (**K**) Graphical summary of the study. During the aging of fly ovaries, Sam-S dependent SAM biosynthesis increases in the germline. Age-related increase in SAM biosynthesis causes age-related oogenesis defects, decrease in GSC number, and increased fused egg chamber formation. Age-related increase in SAM also occurs in mouse reproductive tissues and the brain.

SAM biosynthesis occurs in all tissues in the body and it is evolutionarily conserved between insects and mammalian species. We hypothesized that the age-related changes in SAM biosynthesis occur in mouse tissues. To test this possibility, we analysed the amounts of metabolites involved in SAM biosynthesis in the tissues of young and aged mice. In addition to the reproductive tissues, we analysed the metabolite levels in several parts of the brain (cerebellum, cerebrum, and hippocampus) since it has been shown that the upregulation of the methylation of DNA or histones contributes to the pathogenesis of age-related brain diseases, such as Alzheimer’s disease^8,9^. The level of SAM remarkably increased in the aged tissues (Testis: Fig. 3B, Cerebellum: Fig. 3F, Cerebrum: Fig. 3H, Hippocampus: Fig. 3J), with the exception of the ovary, in which the increase was not significant (Fig. 3D). Therefore, the increase in SAM is an age-related phenomenon common across the species and tissues.

There were differences in SAM-related metabolism between insects and mice. In *Drosophila* ovaries, the SAM content increased in aged ovaries with a decrease in the levels of its precursor Met and its catabolised metabolite SAH. The levels of Met and SAH tended to increase in aged tissues, with some exceptions (Met contents, Testis: Fig. 3A, Ovary: Fig. 3C, Cerebellum: Fig. 3E, Cerebrum: Fig. 3G, Hippocampus: Fig. 3I, SAH contents, Testis: Fig. S1C, Ovary: Fig. S1D, Cerebellum: Fig. S1E, Cerebrum: Fig. S1F, Hippocampus: Fig. S1G), suggesting that the overall Met metabolism, which includes SAM biosynthesis, was enhanced in the tissues of aged mice. In mammals, SAM biosynthesis and homeostasis is crucial for liver function^10^. Alteration in the SAM biosynthesis in the liver is associated with the development of chronic liver disease and hepatocellular carcinoma. The role of SAM biosynthesis in mouse aging needs to be elucidated. To the best of our knowledge, this is the first study that suggests that an increase in SAM is an age-related phenomenon, even in mammalian tissues. Future studies are required to assess if the tissue-specific regulation of SAM biosynthesis influences age-related tissue dysfunction and the longevity of mammals.

Age-related increases in the SAM levels are causal factors that shorten the life span in a variety of species. However, the alteration in SAM biosynthesis during tissue ageing and its influence on tissue aging has not been addressed, because of technical limitations. In this study, we showed that an age-related increase in SAM biosynthesis in the germline was an important causal factor of aging in *Drosophila* oogenesis. SAM is a major cellular donor of the methyl group; and therefore, an increase in the SAM content in the germline could regulate age-related defects by altering the epigenomic status of the germline. Several types of histone methylations, such as methylation at the lysine 4 of histone H3 (H3K4me) and H3K9me, play a crucial role in both GSC maintenance and the development of germline during early oogenesis^11,12^. Therefore, age-related increases in the SAM content in the germline could alter the histone methylation status, causing age-related oogenetic defects. In future studies, it is important to identify the methylation modifications and the regulatory target genes that modulate the aging of oogenesis.

The aging-related increase in the levels of SAH, a downstream metabolite of SAM, is a causal factor in the shortening of the fly life span^13^. SAH accumulates during fly aging and the forced hydrolysis of SAH extends the life span of the fly. Therefore, the enhancement of SAM biosynthesis could cause an accumulation of SAH as the main metabolite causing aging. Our results suggest otherwise, at least in fly oogenesis, because we observed aging-related decreases in the levels of SAH in fly ovaries. Therefore, a tissue-specific downstream causal mechanism of tissue aging could exist. Aging-related increases in the SAH levels were observed in the mouse brain, but not in the reproductive tissues (Figures S1C–G). In mouse brains—similar to that in fly brains—SAH could function as the main metabolite enhancing aging^13^.

The factors causing the aging-related increase in *Sam-S*, which could be the first step of SAM-dependent aging, are not known. In future studies, we will address these points by analysing the aging-related changes in gene expression, epigenomic modification, and Met-related metabolism. Our study provides an important first step in understanding SAM-dependent tissue aging and suggests possibilities for future therapeutic approaches in overcoming aging-related tissue disorders.

## Materials and methods

### Fly strains

Flies were maintained on standard *Drosophila* medium at 25 °C. Germline-specific Gal4 driver *nanos*-Gal4::VP16 (*nos*-Gal4, Gift from D. Van Doren)^14^ was used to express the following constructs: UAS-*Sam-S* RNAi [#36,306 from Bloomington Drosophila Stock Center (BDSC)] and pUASp-*Sam-S* (present study; see below). The *nos*-Gal4 was also crossed with the following strains for the control experiments: Control1, p(CaryP)attP2 (#36,303 from BDSC, control for *Sam-S* RNAi), Control2, and M{3xP3-RFP.attP’}ZH-86Fa (#24,486 from BDSC, control for pUASp-*Sam-S*). These control strains have attP sites for the respective transgenic constructs.

### Mice

Three male and female C57BL/6 mice were used in each experiment. Ten-week-old and eighteen-month-old mice were used as young and old mice, respectively. The mice were housed in home cages in a room that was maintained at 24 °C with illumination, following a 12-h light/dark cycle. Tissue samples were collected after sacrificing the animals through cervical dislocation.

### Immunostaining

Antibody staining was performed according to the standard procedures^15^. Ovaries were dissected from female flies of respective ages in Phosphate Buffer Serine (PBS) and fixed with 4% paraformaldehyde in PBS for 15 min at room temperature. Fixed samples were blocked with 2% bovine serum albumin, 0.1% Tween 20, and 0.1% Triton X-100 in PBS for 30 min. The following primary antibodies were used: mouse anti-Hts antibody [1:5; Developmental Studies Hybridoma Bank (DSHB), 1B1], chick anti-Vasa antibody (1:500; lab stock) and rabbit anti-active Caspase3 antibody (1:500; Abcam, ab13847). For secondary antibodies, we used goat anti-chicken IgY conjugated with Alexa Fluor 488 or 633 (1:500; Thermo Fischer, A-11039, A-21103, respectively) , anti-mouse IgG conjugated with Alexa Fluor 546 (1:500; Thermo Fischer, A-11003) and anti-rabbit IgG conjugated with Alexa Fluor 488 (1:500; Thermo Fischer, A-11008). Primary and secondary antibody incubation was performed overnight at 4 °C. We used a mixture of 2% bovine serum albumin, 0.1% Tween 20, and 0.1% Triton X-100 in PBS an antibody diluent. Stained ovaries were mounted in Vectasheild (Vector Laboratories) and imaged with a confocal microscope (TCS SP5; Leica Microsystems).

### Generation of pUASp-Sam-S transgenic strains

For generating the pUASp-*Sam-S* transgenic strain, PCR-amplified DNA fragments of *Sam-S* were restricted using Kpn I and Xba I and ligated into the pUASK10attB vector^16^. The plasmid construct was inserted into the attP site of the M{3xP3-RFP. attP′}ZH-86Fa strain to generate the pUASp-*Sam-S* transgenic strain.

Primers used for PCR were as follows:

5′-CGGGGTACCTTCAAACTTCGAGTTACATATTAC-′

5′- CGGTCTAGATCAGTTGTCAATCTCCAGAGGCTTG-3′

### Evaluation of GSC and the “fused egg chamber phenotypes” in oogenesis

For GSC counting, we counted the number of the round-shaped cells that had SSs and associated CpCs. For the “fused egg chamber” phenotype, we counted the number of ovarioles that contained at least one germline cyst with more than 16 germline cells. We collected samples from the germline cysts that were in region 3 (or later) of the germaria.

### Metabolite extraction from fly ovaries or mouse tissues

For the extraction of metabolites from fly ovaries, ovaries from 20 female flies were dissected in PBS and collected. The collected samples were immediately frozen and stored at − 80 °C. Metabolites were extracted in 50% methanol, deproteinised using 50% acetonitrile, and dried completely using a centrifugal concentrator (CC-105, TOMY). Pellets were dissolved in 10 mM HCl, followed by filtration using a 0.22-µm polyvinylidene fluoride (PVDF) filter (Millipore). The filtrate was diluted with equal volumes of either 50 mM Tris–HCl (pH 8.8) or 100 µM dithiothreitol. The metabolites from the mouse tissue were extracted and deproteinised in 50% methanol with 10 µM acetic acid. The supernatant was evaporated completely using a centrifugal concentrator (CC-105, TOMY). The pellets were dissolved in Milli-Q water, followed by filtration using a 0.22-µm PVDF filter (Millipore). The precipitates were washed in acetone and the protein was extracted with 0.1 N NaOH at 95 °C for 5 min. The amount of protein was measured using a BCA Protein Assay kit (Thermo Fisher Scientific)^17^.

### UPLC-MS/MS

Ultra-high-performance liquid chromatography equipped with tandem mass spectrometry, TQD (UPLC-MS/MS) (SHIMADZU) was used for measurement of metabolites. The extracted samples were diluted in an equal volume of Milli-Q water. The concentration was calculated based on the standard curve obtained from the serial dilution of the standard solution for each metabolite^18^. Statistical analysis was performed using Rstudio software version 1.4.1717.

### Quantitative-reverse transcription-PCR (qRT-PCR)

For the qRT-PCR of *Sam-S* mRNA, we extracted the RNA from approximately 20 ovaries for the respective age groups using Trizol reagents (Thermo Fisher Scientific). The extracted RNA was then reverse-transcribed using Super Script III Reverse Transcriptase (Thermo Fisher Scientific), and the synthesised cDNA was used as a template for quantitative PCR. We chose the *ribosomal protein 49* (*rp49*) gene as the internal control for this experiment since this gene is broadly used as the internal control for qRT-PCR, even in the case of age-related change of gene expression^2^. Quantitative PCR was performed using the QuantiFast SYBR Green PCR Kit, as per the manufacturer’s instruction and a Rotor-Gene Q thermal cycler (QIAGEN).

Primers used for the qRT-PCR were:

5′-CACGATAGCATACAGGCCCAAGATCGG-3′ and

5′-GCCATTTGTGCGACAGCTTAG-3′ for *rp49*, and

5′-ACAAAATGTGCGACCAAATCAGC-3′ and

5′-CAATCTTTTCGTTTAGTTTGTGAGC-3′ for *Sam-S*.

### Ethical approval

The all the protocols for animal experimentations were carried out in accordance with the approved guidelines in ethical permit approved by the Institutional Animal Care and Use Committee of the National Institute of Advanced Industrial Science and Technology (Permission No. 2021-191) and in accordance with the Law No.105 passed by and the Notification No. 6 released by the Japanese Government. The authors compiled with the ARRIVE guidelines (https://arriveguidelines.org/arrive-guidelines).

## Supplementary Information

Supplementary Figure 1.

Supplementary Figure 2.
