# Supplementary material for: Biosynthesis of S-adenosyl-methionine enhances aging-related defects in Drosophila oogenesis
Source: Sci Rep. 2022 Apr 4;12:5593. doi: 10.1038/s41598-022-09424-1 (PMC8979982; doi:10.1038/s41598-022-09424-1)
Supplement: Supplementary file 1 — Supplementary Figure 1. [file 41598_2022_9424_MOESM1_ESM.docx]

**Figure S1. Age-related changes in SAH levels in fly ovaries and mouse tissues**

(A, B) Age-related change in the SAH levels in the control (A; Control1, B; Control2) and *Sam-S* genetically manipulated (A, *Sam-S*-KD, B, *Sam-S*-OE) ovaries. For multiple comparison analyses in A and B, statistical significance was calculated using Dunnett’s test by using the young control ovaries as the controls; * indicates p < 0.1. (C–G) Age-related change in SAH levels in mouse reproductive tissues (C; Testis, D; Ovaries) and parts of the brain (E; Cerebellum, F; Cerebrum, G; Hippocampus). In C–G, statistical significance was calculated using the Welch’s *t*-test; ** indicates p < 0.05.
