# Supplementary material for: Biosynthesis of S-adenosyl-methionine enhances aging-related defects in Drosophila oogenesis
Source: Sci Rep. 2022 Apr 4;12:5593. doi: 10.1038/s41598-022-09424-1 (PMC8979982; doi:10.1038/s41598-022-09424-1)
Supplement: Supplementary file 2 — Supplementary Figure 2. [file 41598_2022_9424_MOESM2_ESM.docx]

**Figure S2. Germline cell death in germarium.**

(A, B) Germline cell death in the germarium of control2 (A) and *Sam-S*-OE ovaries from young females. In germarium of *Sam-S-OE* ovaries, 36.9% of germarium contained germline cells causing apoptosis labeled by active-Caspase 3 antibody (Green) in contrast to 20.4% of germarium in control2 ovaries (P< 0.05, Significance was calculated with Fisher’s test. The number of ovarioles observed was; Control2: 446, *Sam-S*-OE: 412). Anti-Hts antibody stains fusome (red) and anti-Vasa antibody stains germline cells (Gray). Asterisks indicate GSC and arrowheads indicate germline cells causing apoptosis. Bar; 10µm.
